# Fixation patterns, not clinical diagnosis, predict body size over‐estimation in eating disordered women and healthy controls

**DOI:** 10.1002/eat.22505

**Published:** 2016-03-21

**Authors:** Katri K. Cornelissen, Piers L. Cornelissen, Peter J. B. Hancock, Martin J. Tovée

**Affiliations:** ^1^ Department of PsychologyNorthumbria UniversityTyne and WearUnited Kingdom; ^2^ School of PsychologyStirling UniversityStirlingUnited Kingdom; ^3^ Institute of Neuroscience, Newcastle UniversityTyne and WearUnited Kingdom

**Keywords:** body size over‐estimation, eye‐movements, anorexia nervosa, BMI

## Abstract

**Objective:**

A core feature of anorexia nervosa (AN) is an over‐estimation of body size. Women with AN have a different pattern of eye‐movements when judging bodies, but it is unclear whether this is specific to their diagnosis or whether it is found in anyone over‐estimating body size.

**Method:**

To address this question, we compared the eye movement patterns from three participant groups while they carried out a body size estimation task: (i) 20 women with recovering/recovered anorexia (rAN) who had concerns about body shape and weight and who over‐estimated body size, (ii) 20 healthy controls who had normative levels of concern about body shape and who estimated body size accurately (iii) 20 healthy controls who had normative levels of concern about body shape but who did over‐estimate body size.

**Results:**

Comparisons between the three groups showed that: (i) accurate body size estimators tended to look more in the waist region, and this was independent of clinical diagnosis; (ii) there is a pattern of looking at images of bodies, particularly viewing the upper parts of the torso and face, which is specific to participants with rAN but which is independent of accuracy in body size estimation.

**Discussion:**

Since the over‐estimating controls did not share the same body image concerns that women with rAN report, their over‐estimation cannot be explained by attitudinal concerns about body shape and weight. These results suggest that a distributed fixation pattern is associated with over‐estimation of body size and should be addressed in treatment programs. © 2016 Wiley Periodicals, Inc. (Int J Eat Disord 2016; 49:507–518).

## Introduction

A key diagnostic feature of anorexia nervosa (AN) is a distorted evaluation of personal body size (DSM‐5, 2013), and this is also an important factor in psychological models of the disorder.^1^, ^2^ In treatment, its severity and persistence predicts long‐term outcome and the rate of relapse^2^, ^3^, ^4^, ^5^ which has been estimated to be as high as 35%, 15 months post discharge.^6^ Most studies have found that patients with AN overestimate their body size.^5^, ^7^, ^8^, ^9^ A potential explanation for this may lie in the pattern of eye‐movements made when body size is judged. The estimation of body size is essentially a perceptual task, although cognitive and attitudinal influences may modulate how this percept is interpreted. The physical constraints of the retina mean that detailed information can only be sampled from a small central area of around 2°, corresponding to the fovea.^10^ As a result, the information in a scene can only be collected in small snapshots corresponding to the observer's individual fixations.^11^ Thus the eyes are always moving, sampling the available visual information to construct a representation of the size and shape of a stimulus (such as a body). Some areas of the body (such as the central abdomen) undergo a greater change in size and shape than others (such as the shoulders) when the BMI of a body increases.^12^ Fixating the areas which are sensitive to changing BMI is potentially a good strategy for sampling reliable visual cues to judge BMI. Therefore, a less efficient strategy which samples areas of the body that are less sensitive to weight change could lead to a mis‐estimation of body weight.

A number of studies have compared fixation patterns between women with AN (or women at high risk of eating disorders) and healthy controls. However, although they report differences in the fixation patterns between the women with AN and controls, there is only limited consensus on which areas are favoured or avoided.^13^, ^14^, ^15^, ^16^, ^17^, ^18^, ^19^ This may be due to methodological differences. For example, there is considerable variability in the stimuli used; in their pose, whether the images were in colour or black and white, whether the face or head was shown in the image, the length of the presentation, whether the pictures were of real bodies or of computer‐generated bodies, and in the type and amount of clothing they were wearing.^13^, ^14^, ^15^, ^16^, ^17^, ^18^, ^19^ Perhaps the most important factor in these differences is the choice of the free‐viewing condition for stimulus presentation (i.e., the participants look at bodies without any specific judgement being required of them).^14^, ^16^, ^17^, ^19^ Instead, the eye‐movements are related to the participants' scores on psychometric measures^14^, ^17^, ^19^ or to behavioural judgements which have been made at a different time and not during eye‐movement recording.^16^ This is important because the pattern of fixations is significantly different for different judgements, such as between judgements of attractiveness, body size and body shape.^20^ In a free viewing condition, it is not clear what judgement is being made by a participant. Therefore in this study, we recorded eye‐movements from our participants while they were explicitly making a body size judgement so that we could measure which parts of the body were fixated during that discrimination.

### Study Rationale

In this study, we wanted to separate out eye‐movement patterns that may be specifically associated with the accuracy of body size judgements from those that may be specific to women with AN, but unrelated to the accuracy of their body size estimates. To do this we needed to compare the eye movement patterns from three participant groups: (i) women with AN who have concerns about body shape and weight and who do over‐estimate body size when tested behaviourally, (ii) healthy controls who do not have concerns about body shape but who do over‐estimate body size behaviourally, that is, CN(OVER), (iii) healthy controls who do not have concerns about body shape and who do *not* over‐estimate body size, that is, CN(ACC). A simple comparison between women with AN and CN(ACC), may reveal differences in fixations that produce an over‐estimation in body size estimation or they may be specific to this eating disorder diagnosis. However, if by adding the comparison between AN and CN(OVER) we see the same pattern of differences, we can be confident that these secondary factors can be discounted. This experimental strategy is viable because there is considerable individual variability in the accuracy of body size estimation in control participants.^21^ Consequently, it is possible to identify the critical control group that we need, that is, CN(OVER).

An important consideration is whether differences in the eye‐movements and accuracy of estimation between controls and women with AN might arise as a secondary effect of poor diet and weight loss. Significant functional and structural changes in the brain have been identified due to malnutrition which impact on both cognitive function and ability to control eye‐movements and fixations.^22^, ^23^, ^24^, ^25^ Thus, any simple two‐way comparison between a group of women with AN and a healthy control group, may well be confounded by a range of attitudinal and low level eye movement impairments as a result of their nutritional status. This problem can also be discounted by including the comparison with CN(OVER) controls.

Additionally, recent research shows another source of confounding variation. Cornelissen et al.^20^ have shown that there are linear changes in the *accuracy* of body size estimation as a function of the BMI of the body being judged through two perceptual phenomena; contraction bias^26^ and Weber's Law.^27^ Contraction bias assumes that a body size judgment is made by comparing a body with an internal reference template based on an average of all the bodies that a person has ever seen. This judgement is most accurate when comparing a body similar in size to the internal reference body, and increasingly less accurate as the two diverge.^26^ In these latter cases, when there is an increasing difference between the reference and the body to be estimated, the observer tends to select a response closer to the reference value than it should be. So bodies larger than the reference are underestimated and bodies smaller than the reference are overestimated. Weber's law states that the just noticeable difference (JND) between two stimuli will be a constant proportion of their magnitude, leading to a constant Weber fraction over the stimulus range (i.e., ΔI/I = K, where I = stimulus magnitude and K = constant). This means that, for bodies, it is easier to notice, for example, a one BMI unit difference between two low BMI bodies than between two high BMI bodies. Thus change in the body size will become progressively harder to detect as their BMI increases.^27^ These perceptual biases mean that previous studies comparing body size judgements for women with AN, who have low body weight, versus normal weight controls, may have found differences in accuracy based purely on differences in the sizes of the bodies being judged.

In order to control for both the putative neural effects of malnutrition and the perceptual effects related to BMI, we recruited out‐patients into the study, all of whom have had a diagnosis of AN, but whose BMIs tend to be higher than is typically the case for in‐patients and matched to the BMI of controls. While this means that we controlled for confounding neural and perceptual factors, it also means that our eating disordered group no longer fits the strict DSM‐5 diagnostic criteria with respect to BMI,^28^ although they still have very high body size concerns and eating disordered behaviours. We, therefore, refer to this group as suffering from recovering/recovered anorexia (rAN).

## Methods

The experimental procedures and methods for participant recruitment for this study were approved by: the local ethics committee at Northumbria University; the Beating Eating Disorders Organisation (BEAT) and the Northern Initiative on Women and Eating (NIWE) Organisation. The authors assert that all procedures contributing to this work comply with the ethical standards of the relevant national and institutional committees on human experimentation and with the Helsinki Declaration of 1975, as revised in 2008.

### Participants

Pilot testing showed that the maxima and minima in the group differences in fixations to biologically meaningful areas (e.g., waist and face) could be detected using a sample size of 15 and 18 per group, respectively, (alpha = 0.05 and power = 90%). To offset attrition in participant numbers and/or unexpected sources of variability, we recruited 20 participants per group.

The current study was run in conjunction with a larger scale study, reported in Cornelissen et al.,^21^ in which we investigated the relationship between body size estimation and personal BMI. This meant that we deliberately set out to find female participants with as wide a range of BMI as possible. We recruited from the population of staff and students at Newcastle and Northumbria University and from the general population in and around Newcastle upon Tyne. Potential control participants for the current study were selected initially only on the basis of the fact that when questioned they reported no history of eating disorders. We did not apply other psychometric constraints because we wanted to recruit individuals who are representative of the non‐eating disordered female population, many of whom have concerns about body image (e.g., Ref. 29). We then ran a 2‐alternative forced choice body size estimation task (see below) to identify whether they would be assigned to the over‐estimating, that is, CN(OVER) or the accurately estimating, that is, CN(ACC) control group. The criteria for accurate estimation versus overestimation of body size was a difference between the participant's self‐estimate from psychophysical testing and their actual BMI no greater than +/− 0.7 BMI units versus greater than +1 BMI unit, respectively. These criteria were based, in turn, on psychophysical estimates of the smallest just noticeable difference for BMI for the same stimuli as used in the current study, that is, ∼1 BMI unit.^30^ Control participants were recruited into each group until we had 20 CN(ACC) and 20 CN(OVER) participants.

Participants for the rAN group were recruited if: (i) they originally had a formal diagnosis by a psychiatrist or clinical psychologist of anorexia nervosa according to DSM‐IV‐R or DSM‐5^28^, ^31^ or bulimia nervosa followed by the onset of anorexia—it is not uncommon for patients to move between diagnostic categories.^32^ Note that for the current study we relaxed the BMI criterion, to ensure comparability with the controls (hence the designation of this group as rAN); (ii) they over‐estimated their own BMI by at least 1 BMI unit. Ten of our 20 rAN participants were being treated as out‐patients at the time of testing, while 10 were no longer receiving treatment. We used the permutation method in PROC MULTEST (SAS v9.3) to compute pairwise comparisons between these two subgroups of rAN participants, adjusted for multiple comparisons. There were no statistically significant differences between the two sub‐groups of rAN participants for chronological age, BMI, psychological and perceptual measures. Therefore, henceforth, the rAN group was treated as a single group of individuals for purposes of comparison with the two control groups. In addition, there were no statistically significant differences in BMI between the rAN, CN(ACC) and CN(OVER) groups of participants. However, the rAN group is significantly different from both control groups on all psychological measures. Table 1 details participant characteristics. None of the participants reported other diagnosed psychiatric illnesses.

**Table 1:** Characteristics of the three participant groups: 20 women who have recovered/are recovering from anorexia nervosa—rAN; 20 healthy controls who estimate body size accurately – CN(ACC.); 20 healthy controls who over‐estimate body size CN(OVER). The last three columns show the outcome of pairwise statistical comparisons between groups, controlled for multiple comparisons

Variable	rAN	CN (ACC)	CN (OVER)	rAN v CN (ACC)	rAN v CN (OVER)	CN (ACC) v CN (OVER)
	*M* (*SD*)	*M* (*SD*)	*M* (*SD*)	*p*	*p*	*p*
						
Age	23.70 (4.43)	23.25 (7.93)	20.60 (2.89)	ns	ns	ns
BMI	21.71 (3.95)	23.01 (4.11)	23.19 (5.10)	ns	ns	ns
BDI	26.06 (10.18)	10.47 (6.00)	11.05 (7.22)	*p* < 0.001	*p* < 0.001	ns
EAT	32.68 (15.82)	14.30 (9.37)	11.55 (9.08)	*p* < 0.001	*p* < 0.001	ns
BSQ	70.79 (13.62)	54.45 (18.20)	52.00 (15.02)	*p* < 0.05	*p* < 0.005	ns
EDE‐Q	3.67 (1.40)	2.25 (1.33)	1.96 (1.05)	*p* < 0.05	*p* < 0.001	ns
RSE	11.42 (4.41)	17.53 (4.0)	16.88 (4.83)	*p* < 0.001	*p* < 0.005	ns
PSE – BMI	3.94 (1.96)	−0.07 (0.46)	3.01 (1.19)	*p* < 0.001	ns	*p* < 0.001
DL	0.87(0.81)	0.74(0.37)	1.13(0.78)	ns	ns	ns

Note: BMI = Body Mass Index. BDI = Beck Depression Inventory. EAT = Eating Attitudes Test. BSQ = Body Shape Questionnaire. EDEQ = Eating Disorder Examination Questionnaire global score. RSE = Rosenberg Self‐Esteem Scale. PSE = Point of Subjective Equality. DL = Difference Limen.

### Psychometric and Biometric Measurements

Our experiment design required: (i) participants with recovering/recovered anorexia nervosa (rAN); (ii) that the attitudes of rAN participants towards body shape, weight, and eating were substantially impaired compared to controls; (iii) only body size estimation discriminated between the two control groups. To assess whether participants could fulfil these criteria, they were asked to complete: (1) the 16‐item Body Shape Questionnaire^33^ (BSQ, range 0–96) which indexes the degree of preoccupation and negative attitude toward body weight and body shape; (2) The Eating Attitudes Test^34^ (EAT, range 0–78) which investigates eating habits and dissatisfaction with own body weight and shape. It is a subjective index of the symptoms displayed by individuals with eating disorders and the test is used as a screening questionnaire for eating disorders; (3) The Eating Disorders Examination Questionnaire^35^ (EDE‐Q, range 0–6), which is a self‐report version of the Eating Disorder Examination (EDE) structured interview and measures overall disordered eating behaviour. The participants' level of depression was measured using the Beck Depression Inventory^36^ (BDI, range 0–63) and their self‐esteem was indexed using the Rosenberg Self‐Esteem Scale^37^ (RSE, range 0–30). The participants' body mass index (BMI) was measured with a set of calibrated scales and a stadiometer.

### Psychophysical Measurements

In this study we apply the method of constant stimuli in a 2‐alternative forced choice paradigm. This classical psychophysical method measures two components of the participants' judgements of body size: (i) the point of subjective equality (PSE) which is defined from the psychometric function as the BMI at which participants respond “larger” 50% of the time. Because the PSE represents a complete lack of discrimination, this value corresponds to the body size that participants believe themselves to have; (ii) the difference limen (DL) is the amount of change in a stimulus required to produce a just noticeable difference – in this case between the participant's image of self and the image on screen. The DL has a lower and an upper part. The lower part is the difference in BMI falling between the 25% “larger” response points on the psychometric function and the PSE. The upper part is the difference in BMI falling between the 75% “larger” response points and the PSE. As is commonly the case, we averaged the lower and upper part to give a single estimate of DL. The DL captures the steepness of the psychometric curve and corresponds to how sensitive a participant is to changes in body size.^27^

### Stimulus Image Preparation

**Figure 1:**
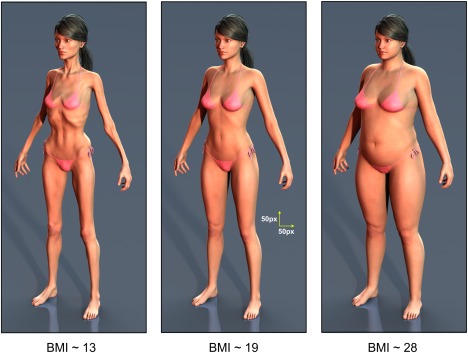
Examples of the body stimuli used in the experiment ranging from the emaciated category to overweight. The central image includes calibration markers to indicate the distances on the stimulus images corresponding to 50 pixels horizontally and 50 pixels vertically. [Color figure can be viewed in the online issue, which is available at wileyonlinelibrary.com.]

We used film industry computer‐generated imagery methods to create graded 3D images of a standard model calibrated for BMI (for details see Cornelissen et al.^21^). The advantages of these stimuli are that the identity of the model in the image is clearly maintained over a wide BMI range and the body shape changes at different BMI levels are realistic (see Fig. 1).

### Psychophysical Procedure

In the 2‐alternative forced choice task, participants were presented with a randomized sequence of images of a standard female body model. Across the image set, BMI varied continuously from 12.5 to 44.5. On each trial of the task, one image was presented and participants were required to decide whether the body depicted was larger or smaller than themselves. Stimuli were presented on a 19″ flat panel LCD screen (1,280 w × 1,024 h pixel native resolution, 32‐bit colour depth) for as long as it took participants to make a decision. At the standard viewing distance of ∼60 cm, the image frame containing the female body subtended ∼26° vertically and ∼8° horizontally. Each participant first judged seven images covering the whole BMI range (from 12.5 to 44.5 in equal BMI steps) presented in two separate blocks. Each stimulus image appeared 10 times in each block, and the order of presentation was randomized. Based on the responses from each block, the participants' point of subjective equality or PSE (the BMI they believe themselves to be) was calculated automatically by fitting a cumulative normal distribution. These two values were then averaged to give an initial estimate of the participant's PSE. On the basis of this initial estimate, the program presented a further set of 21 images (spread over a range of 5 BMI units centred on the participant's initial PSE, at a spacing of 0.25 units per image) for the participants to judge. Each image was presented 10 times in randomized order. This final set of judgements allowed us to plot the full psychometric function (i.e., the proportion of “larger” responses on the *y*‐axis as a function of stimulus BMI on the *x*‐axis) and use probit analysis off‐line to calculate a definitive estimate of PSE as well as the difference limen or DL (that is how sensitive participants are to changes in BMI).

### Eye Movement Recordings

In the eye‐movement condition, the participants completed an abbreviated version of the psychophysical procedure (detailed above) while their eye‐movements were recorded. Participants sat in a dimly illuminated room with their heads supported by a combined head and chin rest. Movements of the right eye were recorded with an Eyelink 1000 eye‐tracker at a sample rate of 1,000 Hz. Stimuli were presented on a CRT monitor at a standard viewing distance of about ∼60 cm. At the start of the session, participants' eye movements were calibrated using a nine point calibration screen. Once the calibration procedure was validated, the experimental task began. We presented eleven stimuli, selected from the same database of 3D rendered images used for psychophysical assessment, which covered the BMI range from ∼12 to ∼45 in equal steps. The experimental task comprised 110 trials (i.e., 11 stimulus images each presented ten times, in random order). The presentation of each body was preceded by a fixation spot, picked at random from one of four locations: top left, bottom left, top right or bottom right of the presentation screen. When the eye‐tracker software had detected that the participant had continuously fixated the fixation spot for 1,000 ms, it was replaced by one of the 11 stimuli which appeared centred on the middle of the screen. The requirement to fixate the fixation spot prevented any anticipatory eye movements. The stimulus remained on screen for as long as it took the participant to decide whether the woman in the image was smaller or larger than they believed themselves to be. The trial ended when the participant pressed the appropriate button to indicate their decision and the next trial was initiated. Behavioural responses from the eye movement recording sessions were treated in a similar fashion to the two‐alternative forced choice task—that is, obtaining each participant's psychometric function by probit analysis off‐line. Note however, that these estimates of PSE are necessarily less refined because they are based only on 11 stimuli covering the full BMI range from ∼12 to ∼45.

The Eyelink 1000 system uses a saccade‐picker approach to identify saccades by applying an exclusive OR rule to three thresholds: velocity (30°/sec), acceleration (8,000°/sec^2^) and distance moved between samples (0.1°). It then treats the rest of the (non‐blink) data as fixations, assuming that the “not in a saccade” condition is maintained for at least 50 ms. The stated accuracy of the system is down to a resolution of 0.15°, though 0.25° to 0.5° is typical.

### Eye Movement Analysis Path

A central problem with eye‐movement recording experiments is how to compare the locations of fixations across successive stimuli of the same class (in this case human bodies) which vary in their size, shape and proportions (such as leg or torso length). A common solution is to pool fixations across relatively large areas of interest (AOIs), each of which represents an anatomically defined sub‐region of the body (such as the right breast, the left hip and so on). However, this approach leads to a considerable loss of spatial resolution and subtle differences in the patterns of fixations between conditions may no longer be detected. Additionally, the same anatomically defined AOI in different images may not occupy the same spatial extent (m^2^) on screen, and so this analysis can introduce a sampling bias into the result, if not controlled for.

To avoid these problems we first morphed all the images of the bodies in our stimulus set together to produce an average or reference body image. This morphing procedure generated a set of coordinate transforms which mapped the individual pixels from each of the original images onto the pixels in the reference image. By applying the same set of transforms to the horizontal (*x*) and vertical (*y*) coordinates of our eye‐movement records, we were able to transform the eye movements for each observer into the same spatial framework and co‐register the fixation patterns with the reference body image. In order to examine the spatial distributions of fixations on the reference body, and to compare fixation patterns across observer groups, we constructed a sampling grid of square cells, 20 × 20 pixels each, and applied it across the entire reference image (1,024 × 574 pixels) (for further details see Ref. 13). This cell size (20 × 20 pixels) represents a compromise between capturing as many fixation samples per cell as possible to optimize statistical power (which ideally requires large cells) versus retaining good anatomical resolution (which ideally requires small cells).

In earlier work^13^ using this analysis method, we showed that the distribution of fixation durations in each cell of the sampling grid is rarely normal, but that the correlation between fixation duration and fixation count is very high (typically *r* > 0.9). Therefore, as before, we chose fixation counts per cell (also known as fixation density) as our outcome variable. We modelled differences in fixation counts between groups by applying generalized linear mixed models (GLMMs). To do this we used PROC GLIMMIX in SAS v9.3 (SAS Institute, NC).

**Figure 2:**
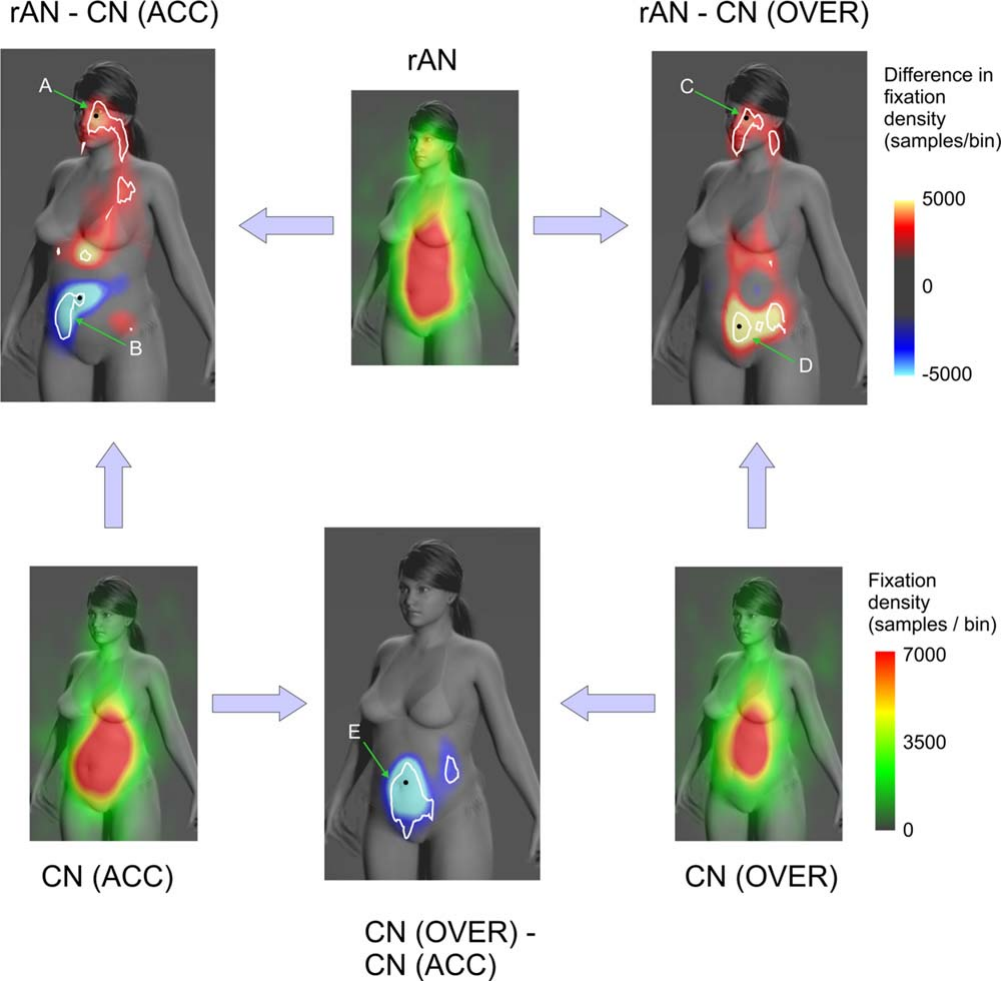
Maps of the relationship between psychophysical performance in body size estimation and where observers look on the body. The colour scales of the heat maps are expressed in terms of fixation counts or differences in fixation counts per sample bin. The heat maps with the green/yellow/red colour scales illustrate the pattern of looking for each of the three groups in isolation. The difference maps with the cyan/blue/red/yellow colour scales illustrate the differences between pairs of groups of participants, indicated by the arrows. Blue/cyan colours indicate where accurate observers looked more frequently than over estimators. Red/yellow colours indicate where over estimators looked more frequently than accurate observers. White lines indicate the regions within which the comparisons between groups were statistically significant at *p* < 0.05 (corrected for multiple comparisons), as defined by the spatial statistical modelling with PROC GLIMMIX (SAS v9.3). The labelled arrows indicate those statistical boundaries for which we report mean fixation duration per subject for each of the groups contributing to a particular comparison (see text for details). The black dots indicate the location of the maximum difference in fixation density for that labelled statistical boundaries.

In each of the three statistical models comparing two groups (i.e., rAN vs. CN(ACC); rAN vs. CN(OVER); CN(OVER) vs. CN(ACC)) we not only took account of the repeated measures factors—that is, each subject contributed a number of fixations to the sampling grid (defined by row and column indices in the model) for each of the 110 images, but we also controlled for spatial co‐variance by incorporating the spatial variability into the statistical models. We assumed the fixation counts to follow the Poisson distribution and consequently a log‐link was used as a link function in the models for the outcome. The spatial variability was integrated into the models by specifying a Gaussian spatial correlation model for the model residuals. The GLIMMIX procedure was then used to assess where on the stimulus images there were significant differences in fixation density between the groups. Areas of significant difference are indicated by the white contours (*p* < 0.05) in Figure 2, and are based on the estimated marginal means derived from the model parameters. These predicted population margins are compared using tests for simple effects by partitioning the interaction effects, and are controlled for multiple comparisons. We also provide descriptions of the extent of these statistical boundaries using anatomical labels and anatomical labelling conventions.

We note that presenting our results in the form of heat maps in Figure 2 has the advantage that we retain good anatomical localization of fixation density, as well as differences in fixation density, onto the body of our reference image. However, interpreting differences in fixation density/count is not necessarily straightforward. Therefore, for purposes of illustration, and to provide a more biologically interpretable sense of effect sizes in the areas where we found the most robust, statistically significant differences, we also report mean fixation durations (i.e., for the boundaries marked A, B, C, D, and E as indicated by the arrows in the difference maps in Fig. 2). To do this, we converted the total fixation density in the set of sampling cells contained within each highlighted statistical boundary into estimates of fixation duration per subject, separately for each group that contributed to the difference. We also marked with a black dot the location of the maximum difference within each of these highlighted the boundaries.

## Results

### Univariate Statistics

The internal consistency of participants' responses to the psychometric questionnaires across the sample was high: Cronbach's alpha for BSQ, EAT, EDE‐Q, RSE, and BDI was: 0.96, 0.92, 0.96, 0.89, and 0.93, respectively.

The right hand columns of **Table** 1 show the output of pairwise comparisons of the group means for the psychometric and psychophysical measures, adjusted for multiple comparisons, using the permutation method in PROC MULTEST (SAS v9.3). **Table** 1 shows that there are no statistically significant differences between the two control groups on any measure, except that, as intended, CN(OVER) and rAN participants over‐estimated their body size whereas CN(ACC) participants did not. **Table** 1 confirms that rAN participants showed significantly elevated concerns about body shape and weight, reduced self‐esteem and increased tendency towards depression compared to both control groups. The mean BSQ score for the rAN participants is above the critical threshold of 66 for eating disorder,^33^ and the mean EDE‐Q global score for this group is above the 90th percentile for young women in the UK^29^ and for female undergraduates.^38^ Both control groups scored within the normal range for the BSQ^33^ and fell within one standard deviation of the population means for women aged 18 to 27 on the EDE‐Q.^29^, ^33^ In addition, both control groups were significantly different from the rAN participants on these measures (**Table** 1). Consistent with the requirement to control for individual BMI, as discussed in the Introduction, the BMI of rAN participants did not differ significantly from either control group. Finally, **Table** 1 confirms that there were no statistically significant differences in task sensitivity (DL) between the three participant groups. Overall, this pattern of performance on the psychometric tasks is consistent with a sample of female participants who have recovered/are recovering from anorexia nervosa and who over‐estimate their body‐size, to be compared with two groups of healthy, non‐eating disordered adult females, one of which over‐estimates body size and the other of which does not.

### Multivariate Statistics

#### Behavioural Responses During Eye Movement Recording

Across all 60 participants, the correlation between PSE estimated from the eye movement recording sessions and the 2‐alternative forced choice task was high (*r* = 0.87, *p* < 0.001). The mean over‐estimation (i.e., PSE‐BMI) computed from responses during the eye movement recordings were: 2.98, −0.46 & 1.41 for rAN, CN(ACC) and CN(OVER) participants respectively. A one way ANOVA with participant group as a factor was statistically significant (F2,57 = 9.65, *p* < 0.001). We found statistically significant post‐hoc comparisons between rAN & CN(ACC) (*t* = 4.39, *p* < 0.001) and CN(ACC) & CN(OVER) (*t* = −2.25, *p* < 0.05), but not between rAN & CN(OVER) (*t* = 1.88, *p* > 0.05). Therefore, despite the fact that the estimates of PSE obtained from the eye movement recording sessions are much coarser than those from the two‐alternative forced choice task, they are nevertheless consistent with each other and confirm that participants performed during the eye movement recordings according to the groups to which they had been they assigned.

The distributions of participants' response times during the eye movement recording sessions were clipped at the mean +/−2 SD separately for each participant. Mean response times for rAN, CN(ACC) and CN(OVER) participants were: 2,505.6, 1,917.4, and 2,354.1 ms, respectively. A one way ANOVA with participant group as a factor was statistically significant (F2,57 = 7.38, *p* < 0.001). Post‐hoc Bonferroni pairwise comparisons between rAN and CN(ACC) and CN(OVER) and CN(ACC) were both statistically significant (*t* = 3.71, *p* < 0.005 and *t* = 2.72, *p* < 0.05, respectively). However, the comparison between rAN and CN(OVER) was not (*t* = 0.94, *p* > 0.05). These results suggest that accurate body size estimation took approximately 1 to 2 fixations less than inaccurate estimation, and critically, that participants appeared to make their decisions after ∼2 to 2.5 s. Consistent with George et al.,^13^ this therefore justified restricting the eye movement analysis to the first 2,000 ms following stimulus onset.

### Eye Movement Data

The analysis of the eye movement data excluded the first 400 ms of each trial and the gaze patterns beyond the time window of 2,000 ms. **Figure 2** shows the fixation patterns for each individual group: rAN, CN(ACC) and CN(OVER) as well as the differences between them. The individual maps show clearly that all participants spend more time looking in the abdominal region than anywhere else. As the fixation spot at the start of each trial appeared randomly in one of the four corners of the screen, this concentration of fixations cannot be explained by the initial fixation position. However, they also show clearly that the tendency to look outside this region, particularly towards the head and chest, was more marked for rAN participants than either CN(ACC) or CN(OVER) participants. These differences, which are apparent on inspection, are confirmed by the statistical group comparisons shown in **Figure 2**. The estimates of r‐square for the three models comparing: rAN vs. CN(ACC); rAN vs. CN(OVER), and CN(OVER) vs. CN(ACC) were 0.62, 0.55 and 0.59, respectively. The white boundaries, indicating regions of statistically significant difference in **Figure 2**, show that rAN participants looked significantly more at the face than did the CN(ACC) group (Fig. 2, boundary A: rAN = 180.7(76.5) ms/subject; CN(ACC) = 48.5(19.1) ms/subject). Anatomically this region of differences includes: left malar and supramedial cheek; left medial canthus; left nasal sidewall; left anterolateral neck. By contrast, rAN participants looked in the central abdominal region significantly less than the CN(ACC) group (**Fig. 2**, boundary B: rAN = 186.2(45.5) ms/subject; CN(ACC) = 381.3(82.3) ms/subject). Anatomically, this difference extends across the left and right umbilical quadrants, and extends inferiorly into the right iliac and hypogastric areas.

The rAN group looked at the face significantly more than the CN(OVER) group (**Fig. 2**, boundary C: rAN = 218.7(77.4) ms/subject; CN(OVER) = 62.2(16.1) ms/subject). This region of differences includes: left malar and supramedial cheek; left medial canthus; left nasal sidewall; upper and lower lips; chin; left anterolateral neck. In addition, the rAN group looked in the abdominal region significantly more than the CN(OVER) group (**Fig. 2**, boundary D: rAN = 459.6(160.5) ms/subject; CN(OVER) = 180.3(46.4) ms/subject). This region of difference includes the hypogastric and left iliac regions. Finally, the CN(OVER) group looked significantly less in the central abdominal region than the CN(ACC) group (**Fig. 2**, boundary E: CN(OVER) = 227.3(49.7) ms/subject; CN(ACC) = 588.2(99.6) ms/subject). This region of difference includes: inferior epigastric, umbilical and hypogastric regions.

To summarize, the comparison of the differences in fixations between the two over‐estimating groups and the accurate controls shows that accurate estimation is associated with fixations concentrated on the lower abdomen, whereas over‐estimation is associated with fixations which are more spread out along the torso and reach up onto the face.

## Discussion

### Is Over‐Estimation Linked to a Specific Pattern of Fixations?

This study compares fixation patterns and the accuracy of body size estimation across three observer groups, two of whom over‐estimate body size and one of whom is accurate. The accurate estimators, CN(ACC), show more fixations than both groups of over‐estimators, CN(OVER) and rAN, in the central and lower abdomen. In contrast, the over‐estimators show more fixations which spread up along the torso and onto the face. The choice of participant groups allows clinical diagnosis, body size concerns, neural deficits and perceptual biases related to BMI to be eliminated as potential alternative causes of the over‐estimation. We find that there is a specific pattern of fixations that is associated with overestimation of body size, and this may either reflect visual sampling of areas of the body which do not give a reliable indication of body mass, or under sampling of those areas that do.

A number of studies have explored how the shape of the body changes with changing body mass. Certain regions of the torso change in proportion to the change in body mass, where others show little change.^39^ Waist circumference is a good predictor of the body's adiposity.^40^, ^41^ For example, Tovée et al.^9^ report that the waist width of 134 adult women is correlated at *r* > 0.90 with their BMI, whereas higher and lower regions of their torso show weaker correlations. Thus, fixations on the central abdomen potentially provide information as to the body's overall adiposity and behavioural experiments have suggested that stomach depth is a strong predictor of judgements of body mass, health and physical attractiveness.^9^, ^39^ By contrast, the relative prominence of the bony landmarks at the top of the torso, like the clavicle and ribcage, may provide information as to how emaciated the body is at low BMIs, but will be less informative for BMI values above this level. (Note the significant differences in the rAN s. CN(ACC) comparison in Figure 2, just below the left clavicle and in the upper epigastrium in the region of the xiphisternum). This interpretation is consistent with a recent study in women recovering from AN, which shows that they are better than controls at estimating the size of very low BMI bodies but are significantly worse than controls at estimating the size of normal and overweight bodies.^21^ As a result, as their BMI increases towards normal levels there is a rapid rise in their over‐estimation of personal body size which may produce a potential pressure for relapse.

### The Role of the Face

A number of studies have suggested that changes in facial shape are correlated with overall body mass and that it is possible to estimate overall body mass from the face alone.^42^ However, the relationship between facial shape and the BMI of the individual is weaker than is found for the equivalent torso shape change, and judgements of overall body mass based on facial cues are also less precise than judgements based on the rest of the body.^42^ This suggests that using the face to judge overall body fat is a suboptimal strategy for estimating body mass and its fixation in preference to the torso may lead to an inaccurate judgement. An alternative explanation is that the eye‐movements towards the face represent a bias toward sampling of socially important information,^43^ although there is some evidence for women with AN actually avoiding faces.^44^ However, although the faces in this study alter in adiposity, no other features (such as expression, gaze direction or identity) change and the participants are familiar with the images and are aware of this. So it seems unlikely that this is an explanation for why our participants with rAN looked more at the faces.

### Potential Confounds in Group Comparisons

In this study we have sought to eliminate possible confounds between our observer groups which might obscure the basis of the body size estimation. The lack of a difference in the BMI of the women in the three groups is important. As we state in the introduction, previous studies have shown that the BMI of the body being judged affects the accuracy of estimation due to contraction bias and Weber's law.^21^ In studies comparing the results of controls and women with AN, the two sets of participants usually have very different BMIs, and as they are estimating the size of their own bodies, the two groups are estimating the size of significantly different bodies. These purely perceptual factors can produce group differences in estimation that may be interpreted as being specific to a clinical group but which would occur whoever was judging a body of that particular BMI. In this study there were no differences in the BMI of the bodies being judged, nor, on average, between observer groups, and so this source of error in estimation was removed.

The neural changes, cognitive impairments and low level motor impairments in eye‐movements that are found in low BMI women with AN as a result of malnutrition largely disappear with refeeding.^22^, ^23^, ^24^, ^25^ Thus, the fixation patterns displayed by the rAN group are unlikely to be due to cognitive impairment or their inability to move their gaze across the image as they would wish. Therefore, the behaviour exhibited by the women with rAN is unlikely to be confounded by motor or cognitive impairment based on malnutrition.

Some previous studies have reported that women with AN look less at the breast region than control observers, a result interpreted as reflecting an avoidance of areas they dislike or have concerns about.^14^, ^16^ This pattern of fixations differs from the results reported here. This could be because one of these studies focused on the perception of attractiveness rather than adiposity,^16^ and there are differences in the pattern of fixations for these two judgements.^13^, ^20^ However, in a previous study we found that when recording the eye‐movements of women with AN making attractiveness judgements, their more distributed pattern of fixations along the torso meant that they looked more at the upper torso than controls.^13^ Another possible reason for this discrepancy is that in these previous two studies, eye‐movements were recorded during a “free‐viewing” of bodies.^14^, ^16^ As their participants were not asked to make a specific judgement during the eye‐tracking, it is not possible to say with certainty what judgement, if any, their participants were making. This might result in differences relative to the fixations made during a specific judgement such as rating bodies for attractiveness or size. However, avoidant behaviour could indeed be an explanation of the more distributed pattern of eye‐movements made by women with AN or rAN. Regions of potential sensitivity, such as the stomach, may be avoided by women with rAN or AN, resulting in a pattern of eye‐movements spread up along the torso as reported here and in our previous study.^13^

### Perceptual versus Cognitive Factors

The disturbance in body size estimation in anorexia nervosa is commonly reported as comprising two components; a perceptual/sensory component and an attitudinal/cognitive component.^4^ The perceptual component is described as an inability to accurately estimate body size. The attitudinal component of body image disturbance consists of dissatisfaction with body shape combined with negative attitudes to weight and shape. Through our between group comparisons we can eliminate this attitudinal component as a source of the over‐estimation, as the over‐estimating controls do not have these concerns. It has been suggested that attitudinal concerns about certain areas of the body (such as the stomach or thighs) drive the differences in eye‐movements between women with AN and controls. There will be a range of performance in any perceptual judgement. Some people will use performance strategies that are less successful than others. It is possible that the women with AN are simply part of the group of people who perform badly on this task. Indeed it is possible that their poor performance on this task and the resultant over‐estimation of body size may have contributed to the development of their disorder. So why does not everybody who has an inefficient pattern of eye‐movements (like the CON(OVER) group) develop significant concerns? Several studies have noted that viewing images of thin bodies can produce body image dissatisfaction, but the development of long‐term and significant body image concerns seems to be limited to a vulnerable subgroup of these women.^45^, ^46^ It is suggested there may be a small subgroup of the population who are predisposed to develop body image concerns and eating disordered behaviour, which can be triggered by environmental conditions. Thus although some women in the general population may just not be very good at estimating body mass, only a subset of them who both over‐estimate and are susceptible to developing eating disorders, will go onto develop significant body image concerns.

Alternatively it is possible that the patterns of eye‐movements made by the CON(OVER) and rAN groups arise from different causes. The pattern of eye‐movements made by rAN may arise from attentional biases or the pattern of fixations may be due to a specialisation for discriminating between very low BMI bodies (such as detecting the bony landmarks). Women with AN spend a great deal of time looking at low BMI bodies including their own, but also online as part of their obsession with the thin ideal.^47^, ^48^ Repeated evaluation and discriminations of low BMI bodies could produce the development of a particular eye‐movement for discriminating between very low BMI bodies. Consistent with this idea, we found that rAN participants tend to look significantly more at faces than either of the two control groups who may simply represent two ends of a normal distribution in size judgement ability. The fixations on the upper chest and face may not be good predictors of overall body fat across the whole BMI range, but the prominence of the underlying bone structure at very low BMI values may provide cues to how thin the body has become.

### Limitations

One limitation of this study is that the bodies used in this experiment are artificial using a simulation of body mass change based on the average pattern of fat deposition rather than real bodies varying in BMI. The advantage of the computer‐generated bodies is that only body mass is changing across the set of stimuli, while skeletal proportions, skin texture and identity are held constant. However, we acknowledge that responding to “virtual” stimuli may engender different patterns of response, compared to stimuli drawn from the real world. Nonetheless the results reported here are consistent with the pattern of fixations made when recording from observers estimating the body size of photographs of real women.^13^, ^20^ Another potential limitation is the inclusion of the face and head in the stimuli, so they were not just body stimuli. However, we would argue that the inclusion of the head makes it a more ecologically valid stimulus (you seldom see bodies without their heads) and features such as the identity, expression, gaze direction and hairstyle are constant across all the images in the stimulus set so the only differences between the faces and heads are the change in adiposity. A final limitation is that the BMI of the women in this study is not below 18.5, which is one of the DSM‐5 criteria for AN. It might be argued that to properly measure the pattern of eye‐movements in an AN population, we should have used a group of women who fully conformed to the DSM‐5 criteria. However, to avoid the perceptual factors linked to observer BMI that confound such a comparison, it would have been necessary to recruit over‐estimating and accurate control groups of the same low BMI. Such individuals are unlikely to have such a low BMI without a medical cause which in turn would introduce new confounding factors into the comparison.

### Treatment Implications

Our results suggest a significant effect of fixation patterns in the judgement of body size, which is unrelated to the psychological or physical state of the observer. If there is a causal link, this suggests that a perceptual training regime to improve the accuracy of body estimation could be an effective adjunct to conventional treatment with cognitive behavioural therapy. One potential way of treating this problem could use a training programme incorporating gaze contingent eye‐tracking (i.e., incorporating a feedback loop from the eye‐tracking to the positioning of the stimulus on the computer monitor) to shift the fixation pattern in women with AN towards the pattern seen in accurate control observers to improve body size estimation accuracy. However, even a simple perceptual training program which gives feedback on body size judgements in a two‐alternative forced choice task can improve body fat judgements in a cohort of women with rAN with a concomitant improvement in their cognitive concerns about body image (Gledhill et al., submitted).

We would like to thank Andre Bester for assistance with programming the eye‐movement task. We would also like to thank the Northern Initiative on Women and Eating (NIWE) for their invaluable help in recruitment.
